# Perinatal nutrition as a key regulator of genomic imprinting: a new paradigm for maternal-child health

**DOI:** 10.3389/fnut.2025.1681847

**Published:** 2025-11-03

**Authors:** Lucia Aronica, Samantha N. Fessler, Emily Stone Rydbom, Randy L. Jirtle

**Affiliations:** ^1^Stanford Prevention Research Center, Stanford University School of Medicine, Stanford, CA, United States; ^2^Needed PBC, Los Angeles, CA, United States; ^3^College of Health Solutions, Arizona State University, Phoenix, AZ, United States; ^4^GrowBaby Life Project, Ashland, OR, United States; ^5^Department of Biological Sciences, North Carolina State University, Raleigh, NC, United States; ^6^Center for Human Health and the Environment, North Carolina State University, Raleigh, NC, United States; ^7^Toxicology Program, North Carolina State University, Raleigh, NC, United States

**Keywords:** epigenetics, genomic imprinting, perinatal nutrition, imprintome, one-carbon metabolism

## Abstract

Genomic imprinting, characterized by parent-of-origin specific gene expression, represents a critical molecular bridge between early life exposures and long-term health outcomes. Unlike most epigenetic marks, inherited gametic imprint control regions normally remain stable across tissues and throughout life, making them valuable biomarkers of early environmental influences. Recent technological advances, particularly the Human Imprintome array, have enabled comprehensive assessment of 1,488 putative imprint control regions (ICRs) that influence development, metabolism, and disease susceptibility, although ongoing experimental validation continues to refine the identification of bona fide ICRs. This perspective explores how maternal and paternal nutrition modifies offspring genomic imprinting patterns with lasting health consequences. We examine evidence from human cohort studies and experimental models demonstrating that periconceptional nutritional status affects methylation at key imprinted regions controlling growth and metabolism. Particular focus is given to one-carbon metabolism nutrients (e.g., folate, vitamin B12, choline) as critical regulators of imprinting establishment and maintenance. We propose that optimizing parental nutrition before and during pregnancy represents a powerful strategy for improving offspring health trajectories by promoting favorable imprinting patterns. The integration of imprintome analysis into maternal care offers unprecedented opportunities for personalized nutritional guidance, and early detection of epigenetic disruptions that may influence lifelong health.

## 1 Introduction

The global burden of maternal and child health complications continues to rise, with environmental exposures and nutritional factors estimated to contribute 70–90% of common chronic diseases in humans (1). While the mechanisms linking early life exposures to later health outcomes are not completely defined, emerging evidence points to perinatal environmentally-induced alterations in the epigenome as being critical in long-term offspring health. Two epigenetically labile subsets of genes that link embryonic environmental exposures with adult disease susceptibility are those that are imprinted and metastable epialleles (2).

Genes with metastable epialleles are established during early development, and have large variability in expression between individuals, but low variability in gene expression between tissues in an individual. The most actively investigated metastable genes are in mice, such as the agouti locus in the agouti viable yellow (A^vy^) mouse (3). Metastable epialleles and correlated regions of systemic interindividual variation (CoRSIV) that control their expression have also been identified in cattle (4) and the human (5), indicating that these systemic epigenetic variants are common in mammals and represent additional targets for nutritional programming alongside imprinted loci. While both metastable epialleles and genomic imprinting represent critical mechanisms linking early environmental exposures to lifelong health outcomes, this perspective focuses specifically on genomic imprinting due to its critical role in human health and disease, the recent availability of comprehensive analytical tools, and the unique stability of imprinting marks across tissues and developmental stages, making them particularly valuable as biomarkers of early life exposures.

Genomic imprinting, characterized by parent-of-origin specific gene expression, regulates 1,488 putative differentially methylated imprint control regions (ICRs) that play essential roles in growth, metabolism, and development (6, 7). While this represents the most comprehensive catalog of candidate ICRs to date, ongoing experimental validation is required to confirm the imprinting status of these regions. Unlike most epigenetic marks that can vary across individuals, tissues, and time, inherited gametic ICRs are normally maintained are maintained with high fidelity across all cells and individuals throughout life in a parent-of-origin dependent manner (8). This stability makes ICRs uniquely valuable as “biological recording devices” that capture early life exposures.

Recent studies have demonstrated that parental nutrition status around conception can dramatically alter methylation patterns at ICRs, with documented effects on offspring growth, metabolism, and disease risk. For example, maternal pre-pregnancy BMI has been associated with altered methylation at multiple ICRs controlling growth and metabolic pathways (9), while gestational diabetes is associated with significant methylation changes at regulatory regions for *GNAS* in infant cord blood and placental tissue that influence energy metabolism and glucose homeostasis (10).

The recent development of the Human Imprintome array represents a technological breakthrough, providing targeted assessment of over 73% of ICRs through a cost-effective platform (11). This innovation enables comprehensive evaluation of how nutritional and environmental exposures impact the human imprintome, and the genomic imprinting landscape of the developing offspring during critical developmental windows. This perspective examines how integrating imprintome analysis into maternal care could enable early detection of environmental impacts and nutritional deficiencies, potentially revolutionizing preventative approaches in maternal-child health. While current evidence suggests that once established, imprinting marks remain largely fixed, nutrition represents a uniquely modifiable factor during the preconception and early gestational periods when these marks are being established and maintained, respectively. The trajectory of nutrition-imprintome research represents a key step toward enhancing focus in research, practice, and policy on nutritional optimization for intergenerational health—empowering parents and healthcare providers with actionable knowledge to influence long-term health outcomes across generations.

## 2 Nutrients as critical modulators of genomic imprinting

### 2.1 Maternal overall nutrition: fundamental regulator of imprinting patterns

The “thrifty genotype” hypothesis suggests that humans evolved to maximize metabolic efficiency during periods of food scarcity. Studies of the Dutch Hunger Winter revealed an epigenetic dimension to this theory—one that operates through genomic imprinting and can be reprogrammed by maternal nutrition within a single generation (12).

When caloric intake was severely restricted to less than 900 calories per day during WWII in the western Netherlands (1944–1945), Tobi et al. identified 181 regions of the genome where DNA methylation was altered by prenatal famine exposure, termed Prenatal malnutrition-associated Differentially Methylated Regions (P-DMRs) (12). These epigenetic changes showed remarkable stability, with methylation differences between exposed and unexposed siblings varying up to 10% and persisting into adulthood.

Individuals exposed during early gestation—when inherited gametic ICRs are maintained by DPPA3, ZFP57, and ZFP445 during the wave of DNA demethylation that occurs soon after fertilization (2, 13, 14)—showed double the rate of type 2 diabetes compared to unexposed siblings, even after adjusting for body mass index. Key P-DMRs were identified at metabolic regulatory genes including INSR and CPT1A, with methylation levels correlating with birth weight and lipid metabolism, respectively. Interestingly, CPT1A (ICR_740) is also a candidate imprinted gene (7). Thus, maternal nutritional restriction during critical developmental windows appears to have induced epigenetic reprogramming that established a thrifty metabolic trajectory, preparing the developing fetus for survival in an anticipated nutrient-poor postnatal environment. However, when these individuals later encountered calorie-dense but nutrient-poor Western diets in adulthood, this adaptive metabolic programming became maladaptive and contributed to increased disease risk.

Individuals who were prenatally exposed to famine during the 1944–45 Dutch Hunger Winter also experienced a doubling in the incidence of the behavioral disorder, schizophrenia (15). This indicates that a major reduction in nutrition during early development markedly increases psychosis formation. Interestingly, the imprinted brain theory, an extension of the conflict theory (16), posits that skewed paternal and maternal expression of imprinted genes results in the behavioral conditions of autism and schizophrenia, respectively (17, 18). Paternally expressed imprinted genes tend to be pro-growth while those that are maternally expressed antigrowth. Consequently, the imprinted brain theory predicts that full term above average-sized babies would have a significantly higher risk of AS disorders concomitant with a significantly lower risk of SS disorders. In contrast, full term below average-sized babies would have a lower risk of AS, but a significantly greater risk for developing SS disorders. This unique opposite risk pattern for AS and SS disorders was indeed demonstrated to be associated with normal variation in birth size in a Danish study of 1.8 million babies of which 95 thousand had AS or SS (19).

Only the imprinted brain extension of the conflict theory of genomic imprinting predicts these opposing neurobehavioral risk patterns, indicating that molecular research on mental disease risk would benefit from nutritional research during early development. Nongenetic factors, such as nutrition in pregnancy, can alter imprinted gene expression by modifying ICR methylation (20). This suggests that the imprinted brain theory may be able to not only explain the notable effect of maternal starvation on risk to develop psychosis, but also the ‘autism epidemic’ of modern Western affluent societies where the diets are calorie-rich (18). Thus, understanding the role of nutrition (18) and environmental factors, like endocrine-disrupting agents (21) in programming both metabolic and neurodevelopmental outcomes through imprinting mechanisms represents a critical frontier in maternal-child health research. Beyond neurodevelopmental outcomes, altered imprinting at the H19/IGF2 locus has also been implicated in other health trajectories. For example, high birth weight is positively associated with an increased risk of breast cancer, particularly among premenopausal women, potentially resulting from increased IGF2 expression due to loss of imprinting at the H19/IGF2 locus (22). These diverse health outcomes underscore the broad and lasting impact of imprinting dysregulation on multiple physiological systems.

### 2.2 One-carbon metabolism: a critical link between nutrition and imprinting

**Figure 1:**
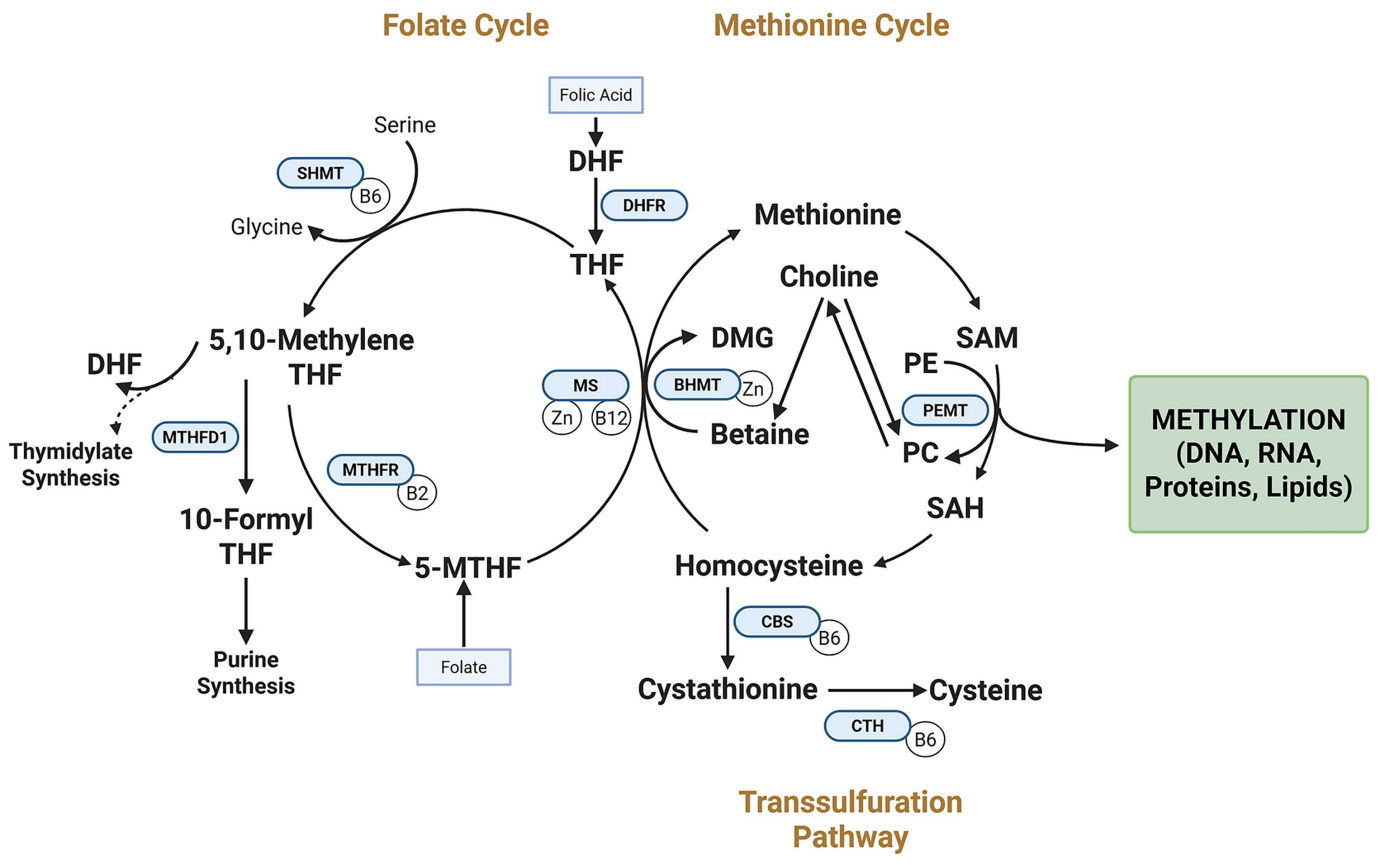
Interrelated pathways of the one-carbon metabolic network and genomic imprinting regulation. The folate cycle, methionine cycle, transsulfuration pathway, and PEMT pathway work together to support S-adenosylmethionine (SAM) synthesis, the universal methyl donor for DNA methylation at imprint control regions (ICRs). Dietary methyl donors (e.g., folate, choline) and cofactors (e.g., vitamin B12) fuel these pathways. Key enzymes (DHFR, MTHFD1, MTHFR) convert folic acid to bioactive forms. Choline contributes methyl groups via betaine and BHMT and supports phosphatidylcholine synthesis via PEMT. The PEMT pathway links choline to phosphatidylcholine synthesis. DHF, Dihydrofolate; SHMT, serine hydroxymethyltransferase; DMG, dimethylglycine; BHMT, betaine-homocysteine S-methyltransferase; Zn, zinc; SAH, S-adenosylhomocysteine; PE, phosphatidylethanolamine; CBS, cystathionine β-synthase; CTH, cystathionine γ-lyase; THF, tetrahydrofolate; 5,10-MethyleneTFH, 5,10-methylenetetrahydrofolate; 10-Formyl THF, 10-formyltetrahydrofolate; 5-MTHF, 5-methyltetrahydrofolate.

The molecular mechanisms linking parental nutrition to offspring imprinting patterns converge on one-carbon metabolism—the essential biochemical pathway that generates methyl groups for DNA methylation (Figure 1). This complex network involves multiple nutrients acting as methyl donors or essential cofactors, including folate, vitamin B12, choline, betaine, and methionine.

The orchestration of one-carbon metabolism represents a sophisticated network of interdependent nutrients that function synergistically rather than in isolation. Evidence increasingly suggests that the epigenetic effects of individual methyl donors depend on the availability and status of complementary nutrients within this metabolic pathway (23, 24). For example, vitamin B12 deficiency can functionally trap folate as 5-methyltetrahydrofolate, impairing remethylation pathways even when folate intake is adequate (25). Similarly, choline can serve as an alternative methyl donor when folate is limited.

In the Gambian studies, maternal blood concentrations of multiple one-carbon metabolites—including folate, vitamin B12, betaine, and choline—collectively predicted offspring methylation patterns better than any single nutrient alone (26). This nutrient synergy has critical implications for nutritional recommendations during preconception and pregnancy, suggesting that optimizing imprinting patterns may require a balanced approach addressing the complete spectrum of methyl donors rather than focusing exclusively on individual nutrients.

While human cohort studies demonstrate associations between maternal nutrient status and offspring ICR methylation, experimental animal studies provide critical mechanistic evidence supporting direct causal relationships. In mice, gestational low protein diet induced loss of imprinting at the Cdkn1c locus through erosion of DNA methylation at the somatic differentially methylated region, effects that were largely prevented by dietary folate supplementation, demonstrating folate’s direct role in maintaining imprinting marks (27). Similarly, both maternal and paternal folate-deficient diets disrupted establishment of GNAS imprinting in mouse gametes and embryos, with aberrant methylation patterns persisting through development (28). Choline deficiency during pregnancy has also been shown experimentally to alter methylation at multiple loci involved in genomic imprinting in offspring tissues (29). These controlled experimental studies establish that one-carbon metabolism nutrients directly regulate imprinting establishment and maintenance, providing mechanistic support for the human epidemiological associations discussed below.

#### 2.2.1 Folate: beyond neural tube defects

While folate’s critical role in preventing neural tube defects has driven public health policy for decades, emerging evidence reveals its broader impact on genomic imprinting and long-term offspring health. As a central methyl donor in one-carbon metabolism, folate availability during critical developmental windows fundamentally shapes the establishment and maintenance of genomic imprinting patterns that persist throughout life.

Folate metabolism directly influences DNA methylation at imprinting control regions (ICRs) through its role in S-adenosylmethionine (SAM) synthesis, the universal methyl donor for DNA methyltransferases (Figure 1). Maternal folate status during early pregnancy affects methylation at key metabolic regulatory regions, with epigenome-wide association studies identifying over 443 significant differentially methylated regions (DMRs) in newborns associated with maternal plasma folate (30).

The timing of folate’s effects appears particularly critical during the periconceptional period when imprinting marks are initially established and maintained (31). Human cohort studies have revealed that maternal folate status in the first trimester correlates with persistent changes in offspring DNA methylation patterns, particularly at genes involved in growth, metabolism, and neurodevelopment (32).

Beyond these considerations, emerging evidence suggests that the chemical form of folate supplementation may also significantly impact imprinting patterns. While folic acid (the synthetic form) and natural folate forms like 5-methyltetrahydrofolate (5-MTHF) both increase conventional biomarkers, the conversion of folic acid to its bioactive form requires several enzymatic steps. These include dihydrofolate reductase (DHFR), methylenetetrahydrofolate dehydrogenase (MTHFD1), and the rate-limiting enzyme methylenetetrahydrofolate reductase (MTHFR). Genetic polymorphisms in these enzymes—particularly the MTHFR variant affecting 40–60% of the population, as well as functional MTHFD1 variants—can alter folate metabolism efficiency. These variants may increase dependence on dietary choline for methyl donation and phosphatidylcholine production (33, 34). Understanding how common genetic variants modulate the relationship between maternal nutrition and offspring imprinting patterns represents an important area for future research.

Unmetabolized folic acid (UMFA) has been detected in over 90% of maternal and cord blood samples in supplemented populations (35), raising concerns about potential deleterious epigenetic effects. Preliminary studies suggest differential effects of folic acid versus 5-MTHF on global DNA methylation patterns, particularly at regions sensitive to nutritional programming (36).

#### 2.2.2 Vitamin B12: the essential cofactor

Vitamin B12 (cobalamin) serves as a critical cofactor in one-carbon metabolism, working synergistically with folate to regulate DNA methylation at imprinted loci. Unlike folate, which directly contributes methyl groups, B12 functions as an essential enzymatic cofactor for methionine synthase (MS), which catalyzes the conversion of homocysteine to methionine (34).

The mechanistic importance of B12 in maintaining genomic imprinting is highlighted by its role in methyl group conservation. B12 deficiency impairs methionine synthase activity, resulting in a “methyl trap” where folate accumulates as 5-methyltetrahydrofolate, but cannot be utilized for methylation reactions (25). This metabolic disruption can significantly reduce the SAM (S-adenosylhomocysteine) ratio, compromising the establishment and maintenance of imprinting marks.

Human cohort studies have revealed that offspring born to mothers with higher serum B12 levels experienced significantly lower weight gain between birth and 3 years compared to those born to mothers with lower B12 levels (37). The *MEG3* DMR appears particularly sensitive to B12-mediated methylation changes. *MEG3* encodes a long non-coding RNA with tumor suppressor properties, and its dysregulation has been implicated in adipogenesis and metabolic disorders (38). Similarly, maternal adherence to a Mediterranean diet—rich in folate, B12, and other methyl donors—has been associated with altered methylation at the MEG3-IG region and improved offspring behavioral outcomes (39, 40).

From a clinical perspective, approximately 10–25% of pregnant women have suboptimal B12 status in developed countries, with even higher rates in regions where vegetarian diets are common (41, 42). Concerningly, high folic acid supplementation can mask vitamin B12 deficiency by correcting the anemia while allowing neurological damage to progress undetected (43). This potential insufficiency represents a significant nutritional gap that may compromise optimal imprinting regulation, particularly in light of increased demands during pregnancy.

#### 2.2.3 Choline: an underappreciated imprinting regulator

Choline stands as a critical, but often overlooked, contributor to epigenetic regulation through its integral role in one-carbon metabolism. As an essential nutrient that serves as both a methyl donor and a precursor to phosphatidylcholine and acetylcholine, choline modulates DNA methylation through multiple pathways.

Human studies have revealed associations between maternal choline status and offspring DNA methylation at specific genomic loci. Jiang et al. (44) examined the effects of third-trimester choline supplementation (930 mg/day versus 480 mg/day) on cord blood and placenta methylation patterns. Higher maternal choline intake led to lower promoter methylation of cortisol-regulating genes and of *GNAS-AS1*, an antisense transcript from the GNAS locus—a well-established imprinted region critical for early development and energy metabolism regulation (44). This higher dose (930 mg)—approximately twice the current choline recommendation in pregnancy—has also been associated with improved neurocognitive outcomes in offspring, with benefits observed even 7 years later in the most recent follow-up data (45).

The adequate intake (AI) for choline increases to 450–550 mg/day during pregnancy, yet surveys consistently report mean intakes below 350 mg/day among pregnant women, with 95% of women not meeting current recommendations (46). This widespread inadequacy represents a significant concern for optimal imprinting regulation.

Interestingly, choline’s effects on epigenetic regulation and neurodevelopmental outcomes exhibit critical period specificity that may differ from other methyl donors. While folate supplementation is typically emphasized preconceptionally and in early pregnancy, emerging evidence suggests choline’s effects may extend throughout pregnancy and even into the postnatal period (47).

Choline also plays a key role in docosahexaenoic acid (DHA) transport through its incorporation into phosphatidylcholine (PC) via the phosphatidylethanolamine N-methyltransferase (PEMT) pathway, potentially linking omega-3 status to methyl donor availability and imprinting maintenance (48). This relationship highlights the complex interconnections between different nutrients in supporting optimal imprinting patterns.

## 3 The human imprintome array: a transformative technology

Understanding the impact of nutrition on the human imprintome (7) has been limited by technical barriers. Existing methylation arrays capture less than 7% of known ICRs and often only at limited CpG sites, while whole genome approaches remain prohibitively expensive. The recent development of the Human Imprintome array provides unprecedented capabilities for comprehensive analysis of imprinting patterns, capturing over 73% of known ICRs at multiple CpGs through a cost-effective platform (11). Unlike most epigenetic marks that vary across tissues and time, the ICRs established during early development are maintained with high fidelity throughout life, making them uniquely valuable for capturing early life exposures. The imprintome array may enable early detection of environmental impacts before complications develop, potentially allowing for identification of risks that might not manifest as clinical symptoms until later in life, but can significantly influence long-term health trajectories.

## 4 Discussion and future directions

Despite the perception that nutritional deficiencies are rare in high-resource settings, women of reproductive age and pregnant women frequently fail to meet current dietary recommendations. Bailey et al. found that 95% of pregnant women in the US would fail to meet dietary recommendations for at least one nutrient through diet alone, with one in three remaining at risk even with supplements (49). These findings highlight the persistent challenge of achieving optimal nutritional status during pregnancy even in high-resource settings.

Nutrition during the periconceptional period and throughout pregnancy represents a powerful modifiable factor for shaping imprinting patterns and long-term health trajectories. The research reviewed here has several important implications:

1 Critical Windows: Evidence suggests that nutritional optimization should begin before conception, as germline imprinting marks are established during gametogenesis, and periconceptional nutrition influences both gamete quality and the maintenance and consolidation of these marks during early embryonic development.
2 Nutrient Synergy: The collaborative action among nutrients in one-carbon metabolism suggests that comprehensive approaches targeting multiple pathways may be more effective than single-nutrient strategies.
3 Personalization Potential: Individual genetic variations affecting nutrient metabolism (such as MTHFR polymorphisms) may influence optimal supplementation strategies, highlighting the need for more personalized approaches.
4 Form Matters: The chemical form of nutrients (such as natural versus synthetic folate) may have differential effects on imprinting regulation, warranting further investigation.

Future research should focus on several key areas:

- Expanding our understanding of how other nutrients, particularly omega-3 fatty acids, vitamin D, and trace minerals, influence imprinting patterns.
- Investigating the paternal contribution to offspring imprinting through nutritional influences on sperm epigenetic marks (50).
- Developing more precise nutritional guidelines that account for individual genetic backgrounds and optimize imprinting patterns for long-term health.
- Leveraging the Human Imprintome array to evaluate how maternal nutritional status impacts the imprintome, potentially revealing new biomarkers and targets for intervention.

By recognizing genomic imprinting as a critical mediator between early nutrition and later health outcomes, we can develop more effective approaches to maternal-child health that address the root causes of common chronic diseases. This represents a paradigm shift: moving beyond reactive disease management to proactively optimizing health potentially across generations with nutritional interventions during early development.

## Data Availability

The original contributions presented in the study are included in the article/supplementary material, further inquiries can be directed to the corresponding author.
